# Supplementary material for: Circular dichroism of quantum defects in carbon nanotubes created by photocatalytic oxygen functionalization
Source: Nat Commun. 2025 Jun 2;16:5107. doi: 10.1038/s41467-025-60342-y (PMC12130195; doi:10.1038/s41467-025-60342-y)
Supplement: Supplementary file 1 — Supplementary Information [file 41467_2025_60342_MOESM1_ESM.pdf]

SUPPLEMENTARY INFORMATION

Circular dichroism of quantum defects in carbon nanotubes created by photocatalytic oxygen functionalization

Finn L. Sebastian[1], *Leon Kaminski*[1], *Christoph Bendel*[2], *Yohei Yomogida*[3], *Yuuya Hosokawa*[4], *Han Li*[5,6], *Sebastian Lindenthal*[1], *Benjamin S. Flavel*[7], *Kazuhiro Yanagi*[4], *and Jana Zaumseil*[1,*]

[1]Institute for Physical Chemistry, Universität Heidelberg, D-69120 Heidelberg, Germany

[2]Institute for Inorganic Chemistry, Universität Heidelberg, D-69120 Heidelberg, Germany

[3]Research Institute for Electronic Science, Hokkaido University, Sapporo, Hokkaido 001-0021, Japan

[4]Department of Physics, Tokyo Metropolitan University, Hachioji, Tokyo 192-0397, Japan

[5]Department of Mechanical and Materials Engineering, University of Turku, FI-20014 Turku, Finland

[6]Turku Collegium for Science, Medicine and Technology, University of Turku, FI-20520 Turku, Finland

[7]Institute of Nanotechnology, Karlsruhe Institute of Technology, D-76131 Karlsruhe, Germany

Corresponding author

*E-mail: zaumseil@uni-heidelberg.de

CONTENTS

Supplementary Figures **3**
Suppl. Fig. 1 | Characterization of pristine (6,5) SWCNTs **3**
Suppl. Fig. 2 | LED output and AQS absorption **4**
Suppl. Fig. 3 | SWCNT functionalization tracking **5**
Suppl. Fig. 4 | Reference experiments I **6**
Suppl. Fig. 5 | Reference experiments II **6**
Suppl. Fig. 6 | Raman spectroscopy of SWCNTs **7**
Suppl. Fig. 7 | TCSPC measurements **8**
Suppl. Fig. 8 | Excitation density-dependent PL **9**
Suppl. Fig. 9 | Temperature-dependent PL **10**
Suppl. Fig. 10 | Different surfactants **11**
Suppl. Fig. 11 | Absorption spectra of additional nanotube species **12**
Suppl. Fig. 12 | PLE maps of pristine SWCNTs **13**
Suppl. Fig. 13 | PLE maps of functionalized SWCNTs **14**
Suppl. Fig. 14 | Optical trap depths for different SWCNT species **15**
Suppl. Fig. 15 | SWCNT dispersions in PL-PEG **16**
Suppl. Fig. 16 | SWCNTs wrapped by ssDNA **17**
Suppl. Fig. 17 | Functionalization of CoMoCAT SWCNTs **18**
Suppl. Fig. 18 | Tip-sonicated SWCNTs **19**
Suppl. Fig. 19 | AFM length statistics **20**
Suppl. Fig. 20 | Organic solvent SWCNT dispersions **21**
Suppl. Fig. 21 | PLE maps of organic solvent SWCNT dispersions **22**
Suppl. Fig. 22 | Treatment of (6,5) SWCNTs with *t*-Bu-AQ in toluene **23**
Suppl. Fig. 23 | Single-nanotube PL spectra at cryogenic temperature **24**
Suppl. Fig. 24 | Spatial resolution of film functionalization **25**
Suppl. Fig. 25 | Absorption spectra of SWCNT enantiomers **26**
Suppl. Fig. 26 | PLE maps of SWCNT enantiomers **27**
Suppl. Fig. 27 | UV-Vis CD spectra of functionalized SWCNTs **28**
Suppl. Fig. 28 | Raman spectra of SWCNT enantiomers **29**
Supplementary Note 1 .... **30**
Supplementary References **32**

Supplementary Figures

Suppl. Fig. 1 | Characterization of pristine (6,5) SWCNTs

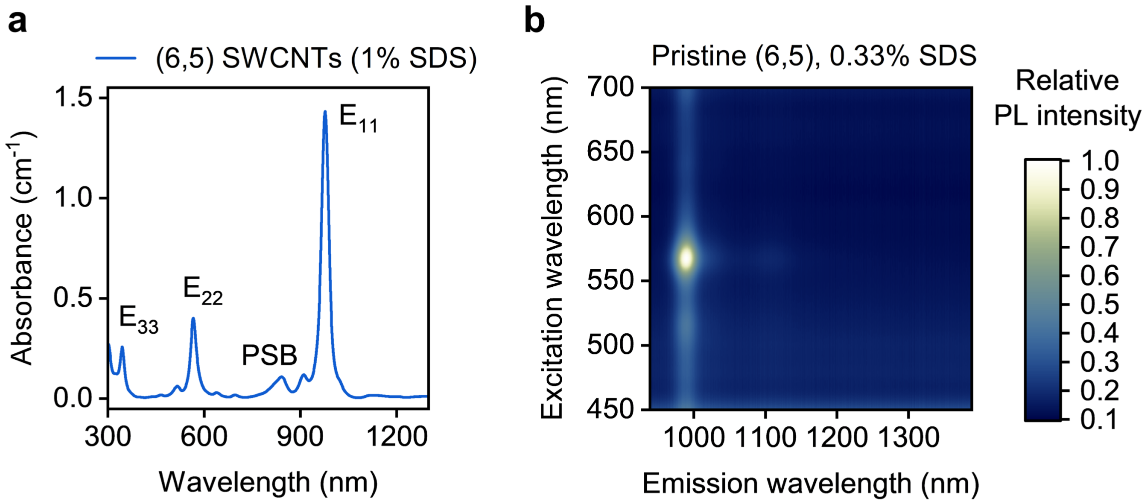

Supplementary Fig. 1 | Characterization of pristine (6,5) SWCNTs. a UV-Vis-NIR absorption spectrum of a pristine (6,5) SWCNT dispersion in H_2O (1% w/v SDS) and assignment of main absorption features (E_{ii} transitions and phonon side band (PSB)). **b** PL excitation-emission (PLE) map of a diluted dispersion (0.33% w/v SDS).

Suppl. Fig. 2 | LED output and AQS absorption

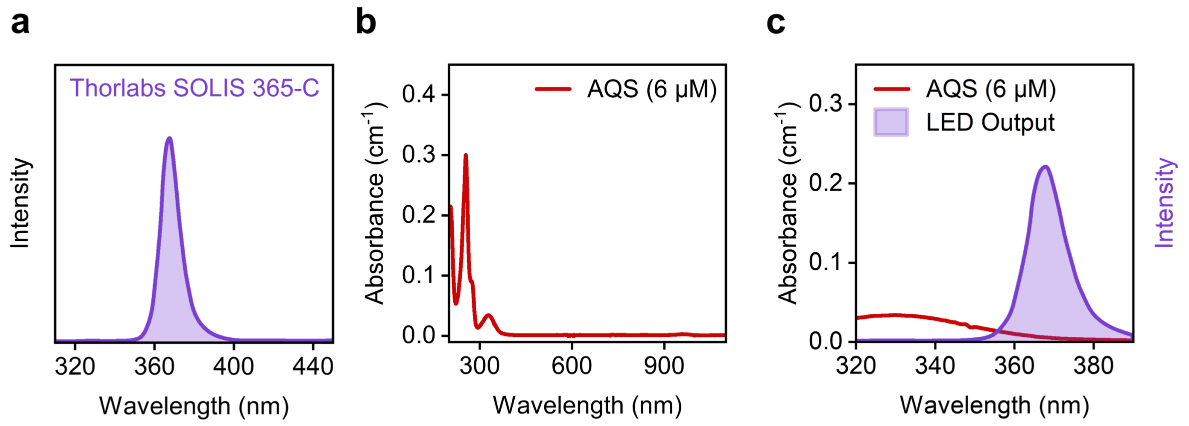

Supplementary Fig. 2 | LED output characteristics and UV-Vis absorption spectrum of AQS. **a** Spectral output of the LED employed for the photocatalytic functionalization reaction (Thorlabs SOLIS 365-C). **b** UV-Vis-NIR absorption spectrum of anthraquinone-2-sulfonate (AQS) at identical concentration as used for the functionalization of (6,5) SWCNTs in H_2O. **c** Overlay of the absorption spectrum of AQS and the spectral output of the LED (note that the scaling of the LED output intensity was chosen arbitrarily in reference to the absorption spectrum to highlight the spectral overlap).

Suppl. Fig. 3 | SWCNT functionalization tracking

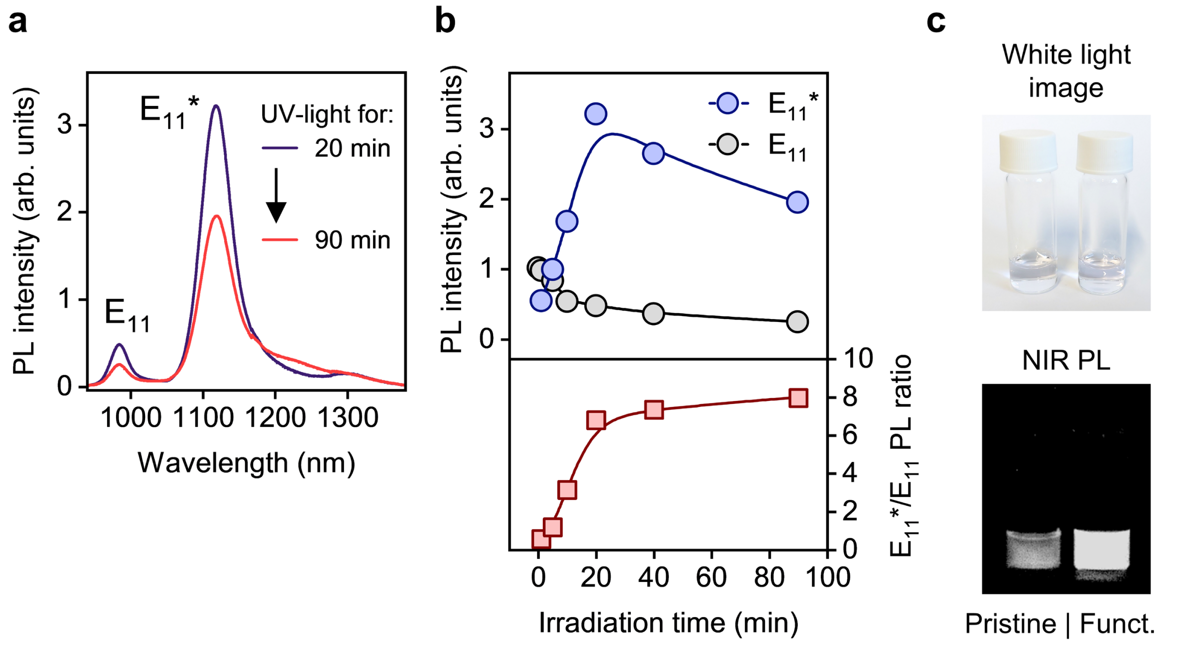

Supplementary Fig. 3 | Tracking of the photocatalytic functionalization reaction and NIR PL imaging. a Reduction in absolute PL intensity under further irradiation with UV-light (365 nm) after absolute E_{11}* PL emission intensity maximum was reached. **b** Evolution of the absolute and relative E_{11} and E_{11}* PL intensities with increasing duration of UV-light irradiation (lines are a guide to the eye). **c** White light image and NIR PL image (950 – 1600 nm, excitation with 525 nm LED) of pristine and functionalized (6,5) SWCNT dispersions (0.33% w/v SDS) at identical concentration.

Suppl. Fig. 4 | Reference experiments I

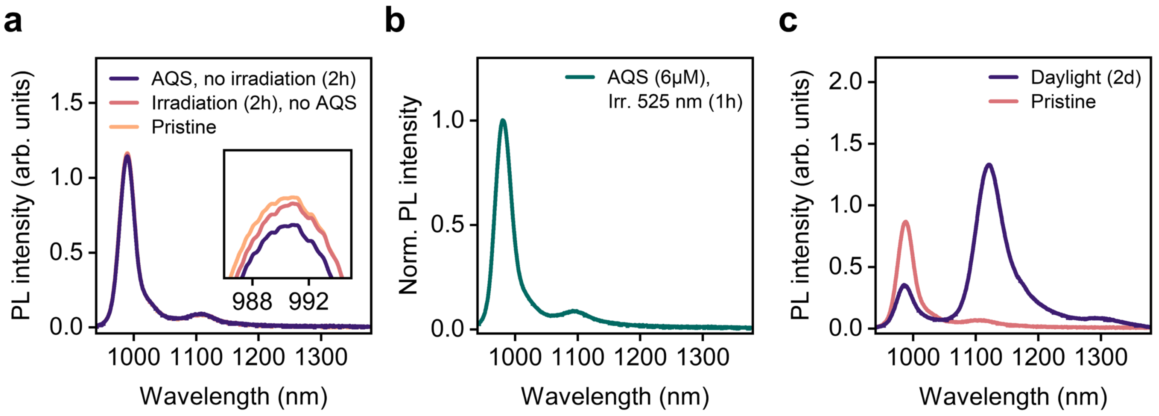

Supplementary Fig. 4 | a PL spectra of a pristine aqueous (6,5) SWCNT dispersion (0.33% w/v SDS), the same dispersion 2 h after addition of AQS (6 µM) but without irradiation, and dispersion 2 h after continuous irradiation (365 nm LED) but without prior addition of AQS. **b** PL spectrum of (6,5) SWCNT dispersion after addition of AQS (6 µM) and continuous irradiation with a 525 nm LED (Thorlabs SOLIS 525-C), showing no signs of functionalization. **c** Limited functionalization after exposure to daylight for 2 days (6 µM AQS).

Suppl. Fig. 5 | Reference experiments II

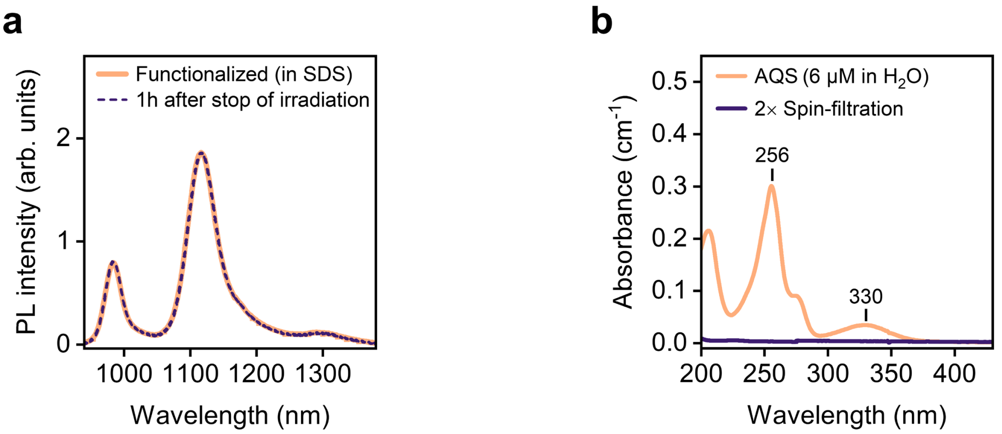

Supplementary Fig. 5 | a PL spectra of a functionalized (6,5) SWCNT dispersion (in 0.33% w/v SDS), directly after stopping UV-light irradiation and 1 h later. **b** UV-Vis absorption spectra of an aqueous AQS solution as it is used in the functionalization procedure (concentration 6 µM) and after the standard work-up of functionalized SWCNT dispersions (two consecutive rounds of spin-filtration), showing no residual AQS in the dispersion.

Suppl. Fig. 6 | Raman spectroscopy of SWCNTs

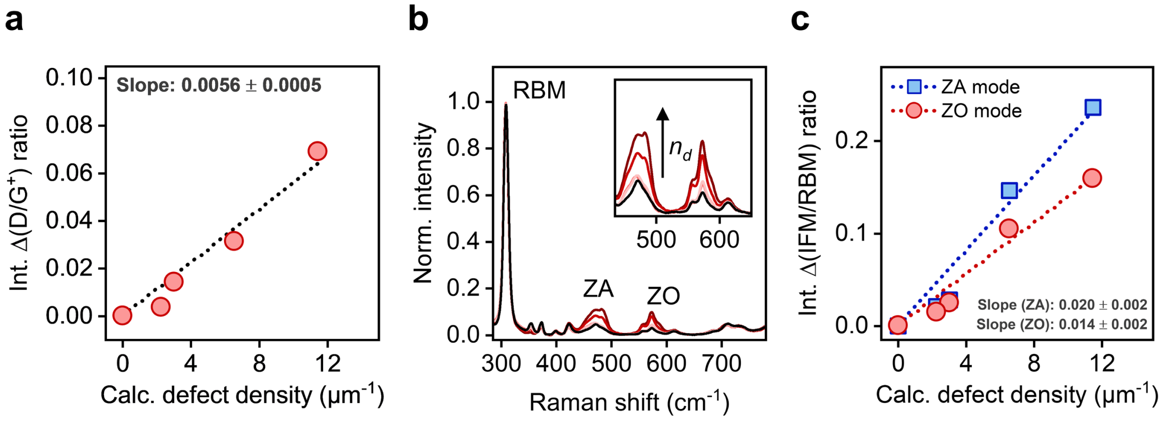

Supplementary Fig. 6 | Raman spectroscopy of pristine and functionalized (6,5) SWCNTs.
a Correlation of integrated Raman Δ(D/G^+) ratios vs calculated defect density for functionalized (6,5) SWCNTs as depicted in Figure 1d (main text) and linear fit to the data (R^2 = 0.97). **b** Averaged and normalized Raman spectra of pristine and functionalized (6,5) SWCNTs in the RBM and IFM region (λ_{exc} = 785 nm). **c** Correlation of integrated Raman Δ(IFM/RBM) ratios vs calculated defect densities (linear fits to the data, R^2(ZA) = 0.97, R^2(ZO) = 0.97)[1,2].

Suppl. Fig. 7 | TCSPC measurements

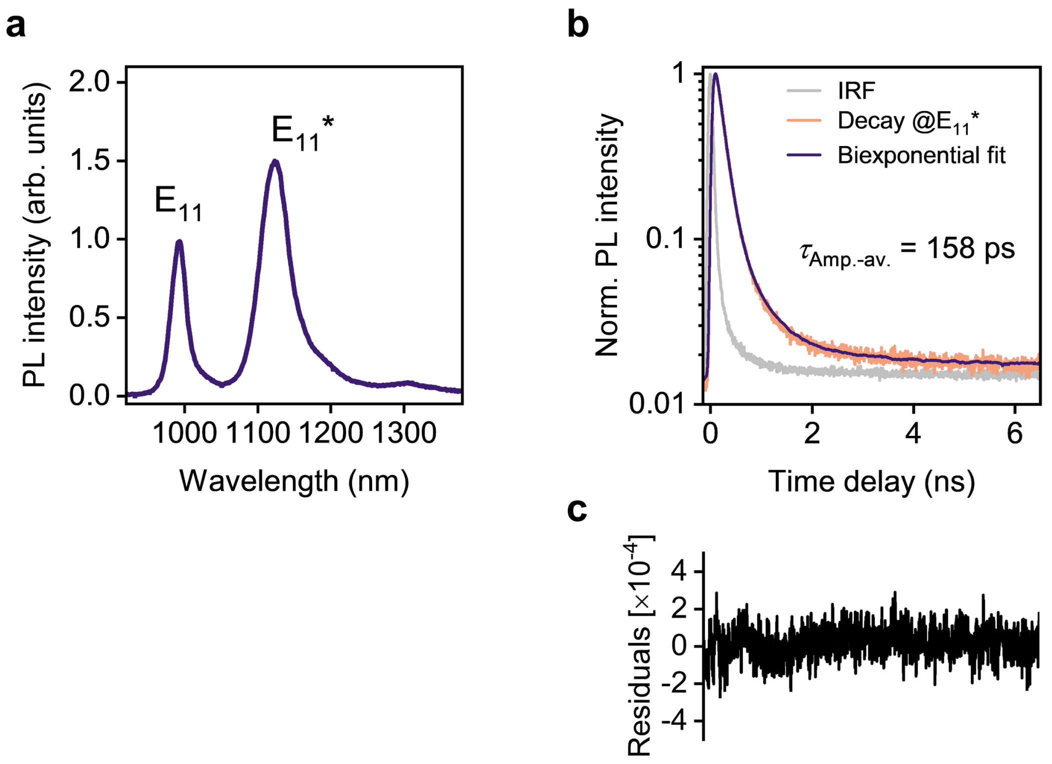

Supplementary Fig. 7 | Time-resolved photoluminescence measurements. a PL spectrum of aqueous (6,5) SWCNT dispersion used for time-resolved PL measurements. **b** TCSPC (time-correlated single-photon counting) histogram of the E_{11}* PL decay measured at 1120 nm (orange) and biexponential fit to the data (dark blue) according to Hartmann et al.[3] The instrument-limited decay of the E_{11} emission was used to obtain the instrument-response function (IRF, gray). An amplitude-averaged lifetime ($\tau_{Amp.-av.}$) of 158 ps was extracted. **c** Corresponding residuals of the biexponential fit from **b**.

Suppl. Fig. 8 | Excitation density-dependent PL

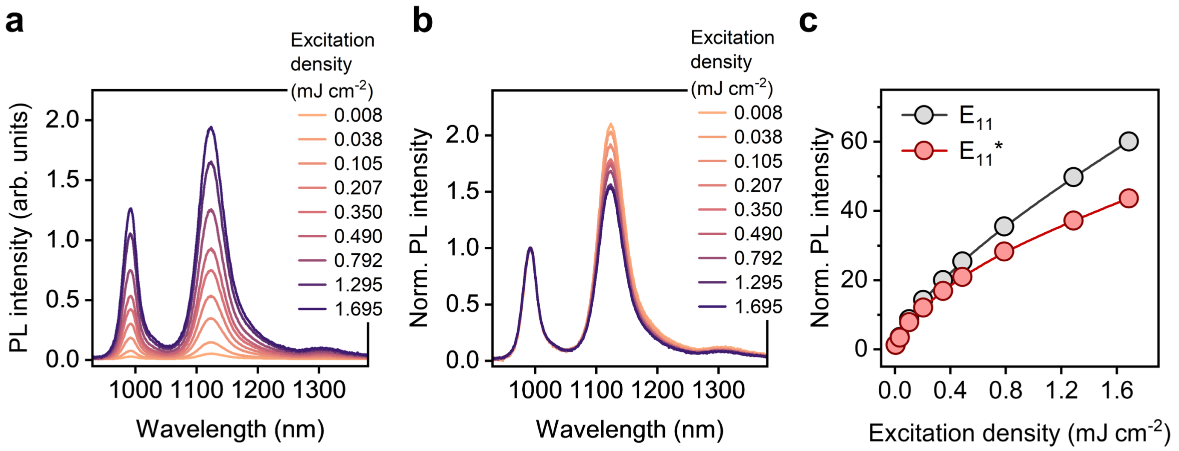

Supplementary Fig. 8 | Dependence of E_{11} and E_{11}* PL emission on excitation density. a PL spectra of oxygen-functionalized (6,5) SWCNTs (aqueous dispersion, 1% w/v DOC) at different laser excitation densities (pulsed excitation at E_{22}, λ_{exc} = 570 nm). **b** Normalized (to E_{11}) PL spectra at different laser excitation densities. **c** Intensities of E_{11} (gray) and E_{11}* (red) emission normalized to their respective value at the lowest laser excitation density, lines are guides to the eye.

Suppl. Fig. 9 | Temperature-dependent PL

The thermal detrapping energy ΔE_{therm} can be extracted from temperature-dependent PL measurements of functionalized SWCNTs using van't Hoff plots as introduced by Kim et al.[4] This analysis assumes reversibility of exciton trapping at defect sites if the thermal energy kT exceeds the depth of the defect state potential. Thus, the relative PL intensity of the E_{11} and E_{11}* emission exhibits a dependence on the temperature according to:

$$\frac{A_{E_{11}}}{A_{E_{11}^*}} \propto \exp\left(-\frac{\Delta E_{\text{therm}}}{\text{k}T}\right) \tag{2}$$

$$\ln\left(\frac{A_{E_{11}}}{A_{E_{11}^*}}\right) \propto -\frac{\Delta E_{\text{therm}}}{\text{k}T} + C \tag{3}$$

Integrated PL intensities are denoted by $A_{E_{11}}$and $A_{E_{11}^*}$; k is the Boltzmann constant, T the absolute temperature, and C is a correction factor. PL spectra of functionalized (6,5) SWCNTs were acquired in a temperature range from 283 K to 328 K in increments of 5 K. Integrated PL intensities were extracted from normalized spectra (see Suppl. Fig. 9a, b). A linear fit according to Equation (2) yields ΔE_{therm} = 102.4 meV (see Suppl. Fig. 9c).

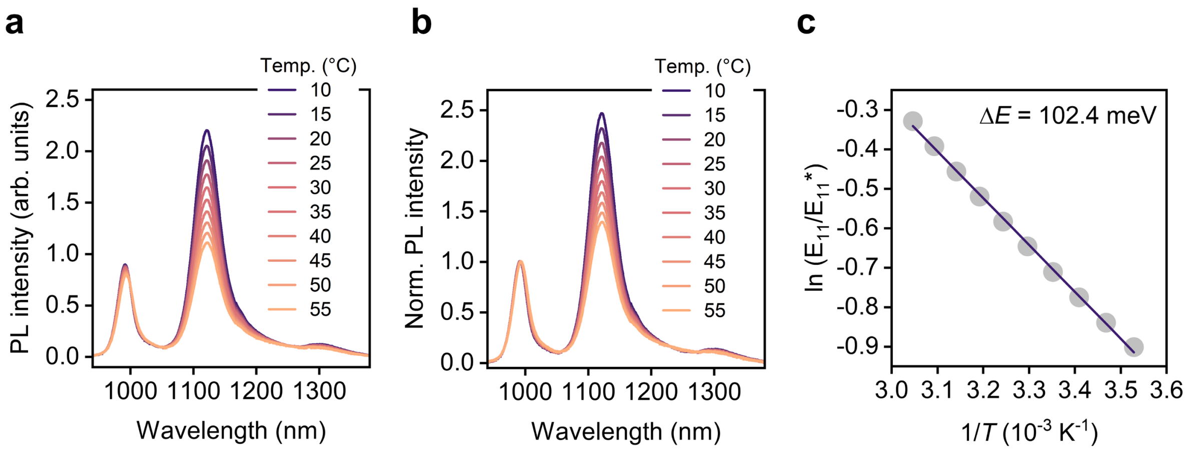

Supplementary Fig. 9 | Temperature-dependent photoluminescence measurements. a Temperature-dependent (283 K to 328 K) PL spectra of oxygen-functionalized (6,5) SWCNTs (aqueous dispersion, 1% w/v DOC). **b** Corresponding normalized (to E_{11}) PL spectra. **c** van't Hoff plot for the E_{11}* emissive state, linear fit to the data (R^2 = 0.99) and extraction of a thermal detrapping energy ΔE_{therm} = 102.4 meV.

Suppl. Fig. 10 | Different surfactants

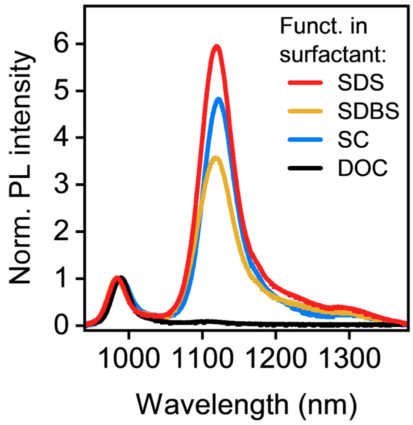

Supplementary Fig. 10 | Functionalization in dispersions with different surfactants. Photocatalytic functionalization (6 µM AQS, 20 min UV irradiation) of (6,5) SWCNTs stabilized by different surfactants (concentrations: SDS 0.33% (red), SDBS 0.33% (orange), SC 0.80% (blue), DOC 0.33% (black), all in (w/v)).

Suppl. Fig. 11 | Absorption spectra of additional nanotube species

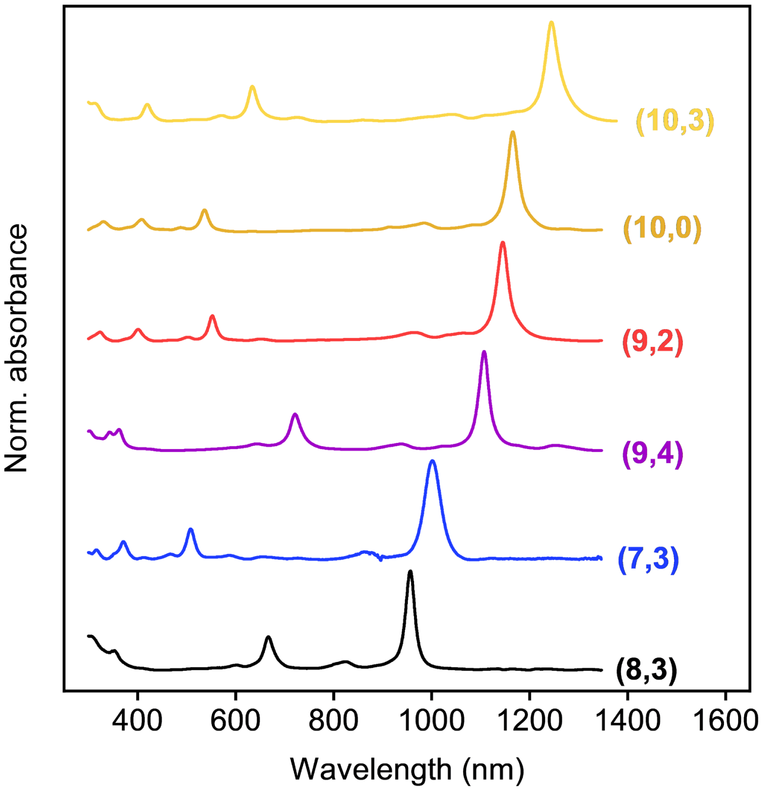

Supplementary Fig. 11 | Absorption spectra of different SWCNT species. UV-Vis-NIR absorption spectra of sorted (10,3), (10,0), (9,2), (9,4), (8,3), and (7,3) SWCNT dispersions before functionalization (in 0.33% w/v SDS). E_{22} optical transition wavelengths for optical excitation in PL spectroscopy were determined from the depicted spectra: (8,3) – 668 nm, (7,3) – 509 nm, (9,4) – 723 nm, (9,2) – 554 nm, (10,0) – 538 nm, (10,3) – 636 nm.

Suppl. Fig. 12 | PLE maps of pristine SWCNTs

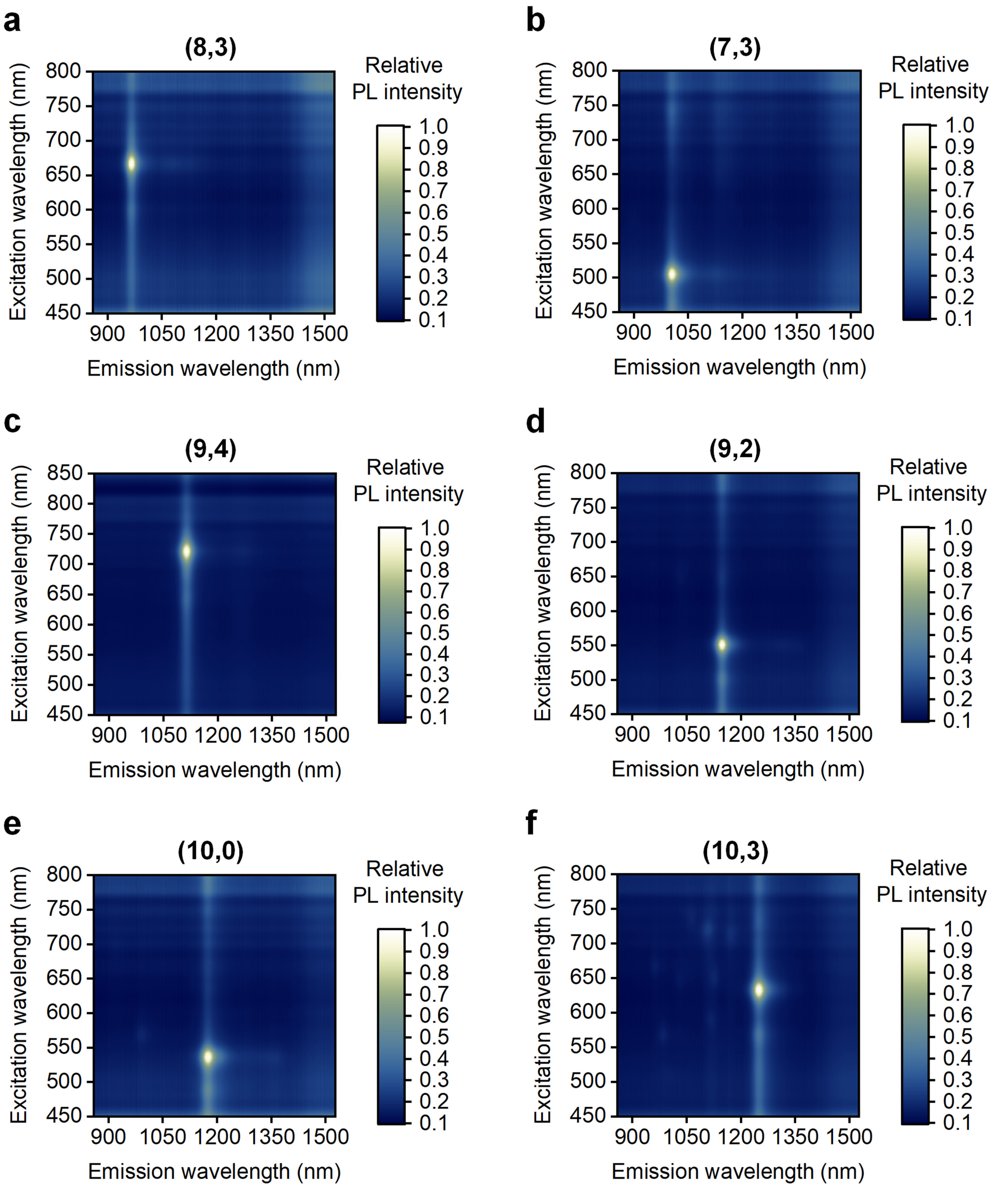

Supplementary Fig. 12 | PLE maps of different pristine SWCNT species. Aqueous dispersions (0.33% w/v SDS) of (8,3) (**a**), (7,3) (**b**), (9,4) (**c**), (9,2) (**d**), (10,0) (**e**), and (10,3) (**f**) SWCNTs.

Suppl. Fig. 13 | PLE maps of functionalized SWCNTs

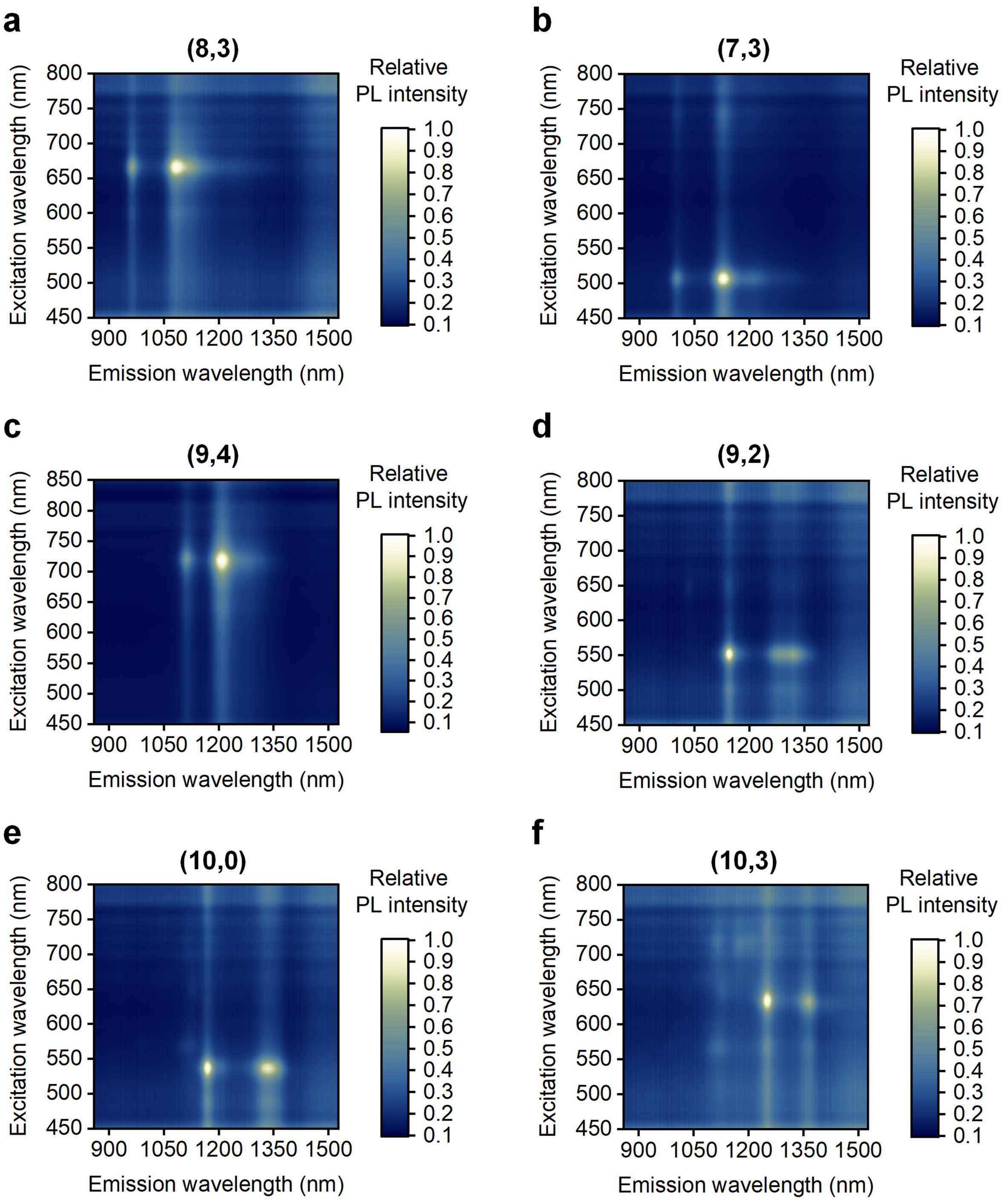

Supplementary Fig. 13 | PLE maps of different functionalized SWCNT species. Aqueous dispersions (0.2% w/v DOC) of (8,3) (**a**), (7,3) (**b**), (9,4) (**c**), (9,2) (**d**), (10,0) (**e**), and (10,3) (**f**) SWCNTs.

Suppl. Fig. 14 | Optical trap depths for different SWCNT species

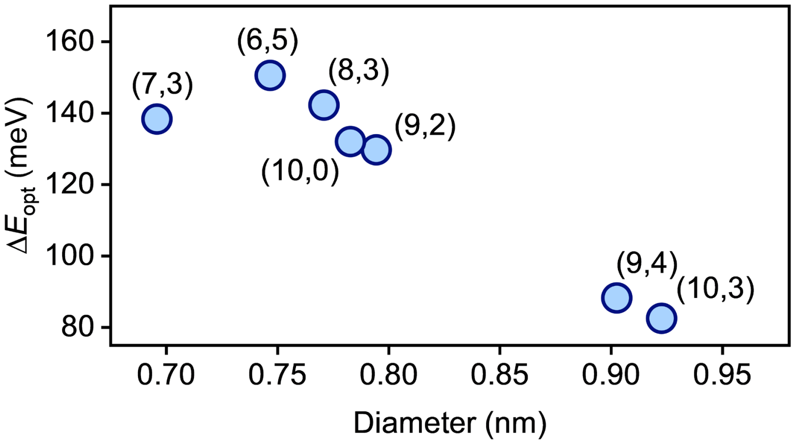

Supplementary Fig. 14 | Diameter dependence of optical trap depth. Optical trap depths ΔE_{opt} vs SWCNT diameter (data extracted from E_{11} and E_{11}* PL peaks of different oxygen-functionalized SWCNT species, see Fig. 2c, main text).

Suppl. Fig. 15 | SWCNT dispersions in PL-PEG

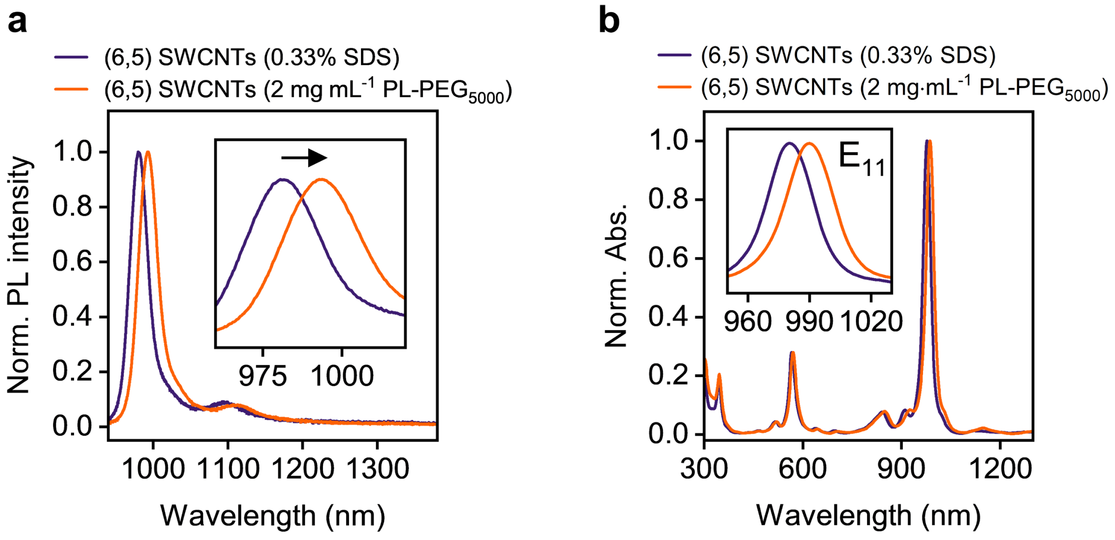

Supplementary Fig. 15 | Transfer of (6,5) SWCNTs to PL-PEG$_{5000}$. a Normalized PL spectra of (6,5) SWCNTs before and after exchange of surfactant system from SDS (0.33% w/v) to PL-PEG$_{5000}$ (2 mg mL^{-1}). The successful exchange to PL-PEG$_{5000}$ is indicated by a redshift (≈ 13 nm) of the E_{11} peak. **b** UV-Vis-NIR absorption spectra of (6,5) SWCNTs before and after transfer to PL-PEG$_{5000}$.

Suppl. Fig. 16 | SWCNTs wrapped by ssDNA

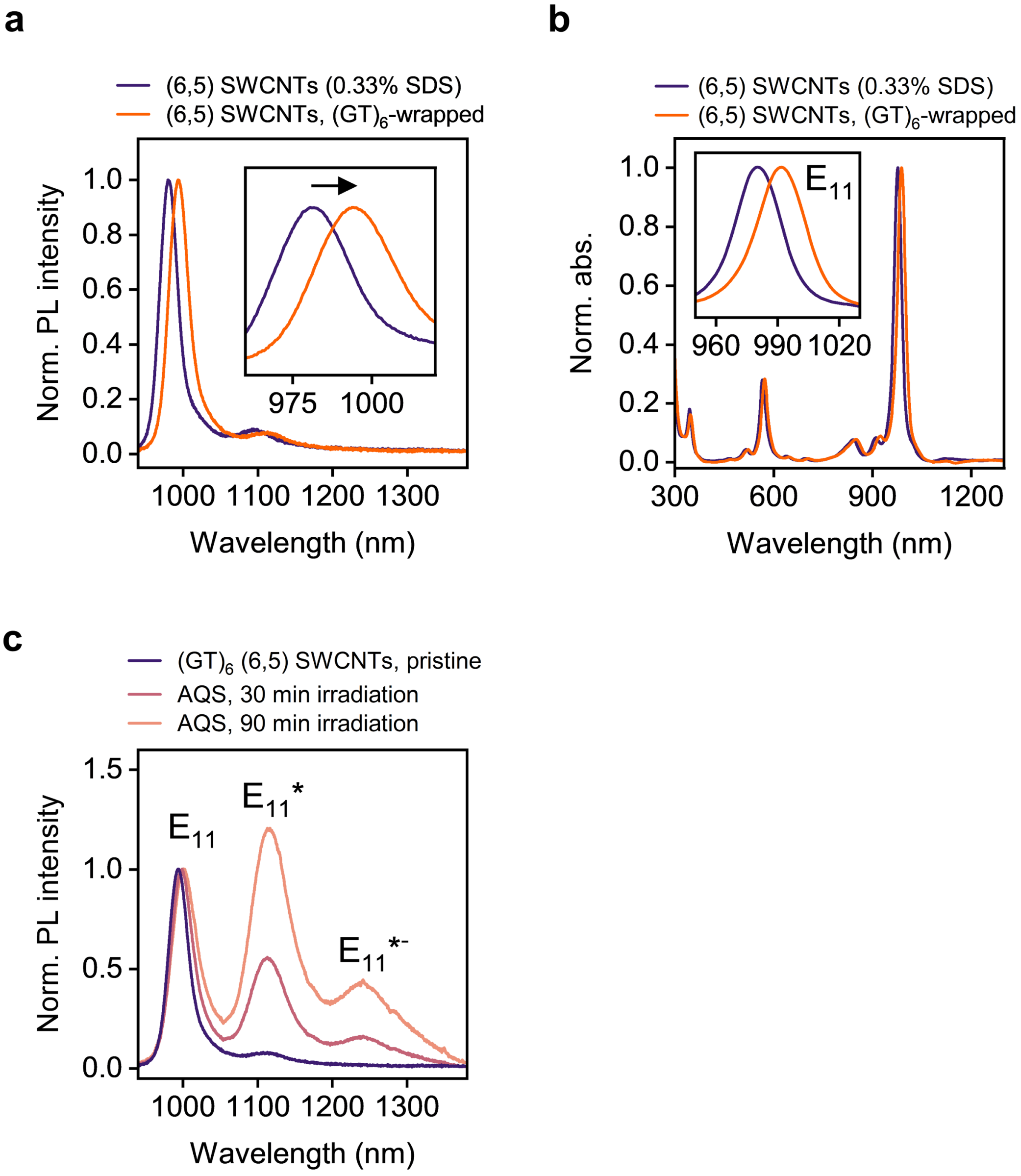

Supplementary Fig. 16 | Functionalization of ssDNA-wrapped SWCNTs. a PL spectra of pristine (6,5) SWCNTs before and after transfer from SDS surfactant to $(GT)_6$-ssDNA, indicated by a redshift of ≈ 14 nm. **b** UV-Vis-NIR absorption spectra of (6,5) SWCNTs before and after transfer to $(GT)_6$-ssDNA. **c** Photocatalytic functionalization of $(GT)_6$-wrapped (6,5) SWCNTs using AQS in 1× PBS buffer.

Suppl. Fig. 17 | Functionalization of CoMoCAT SWCNTs

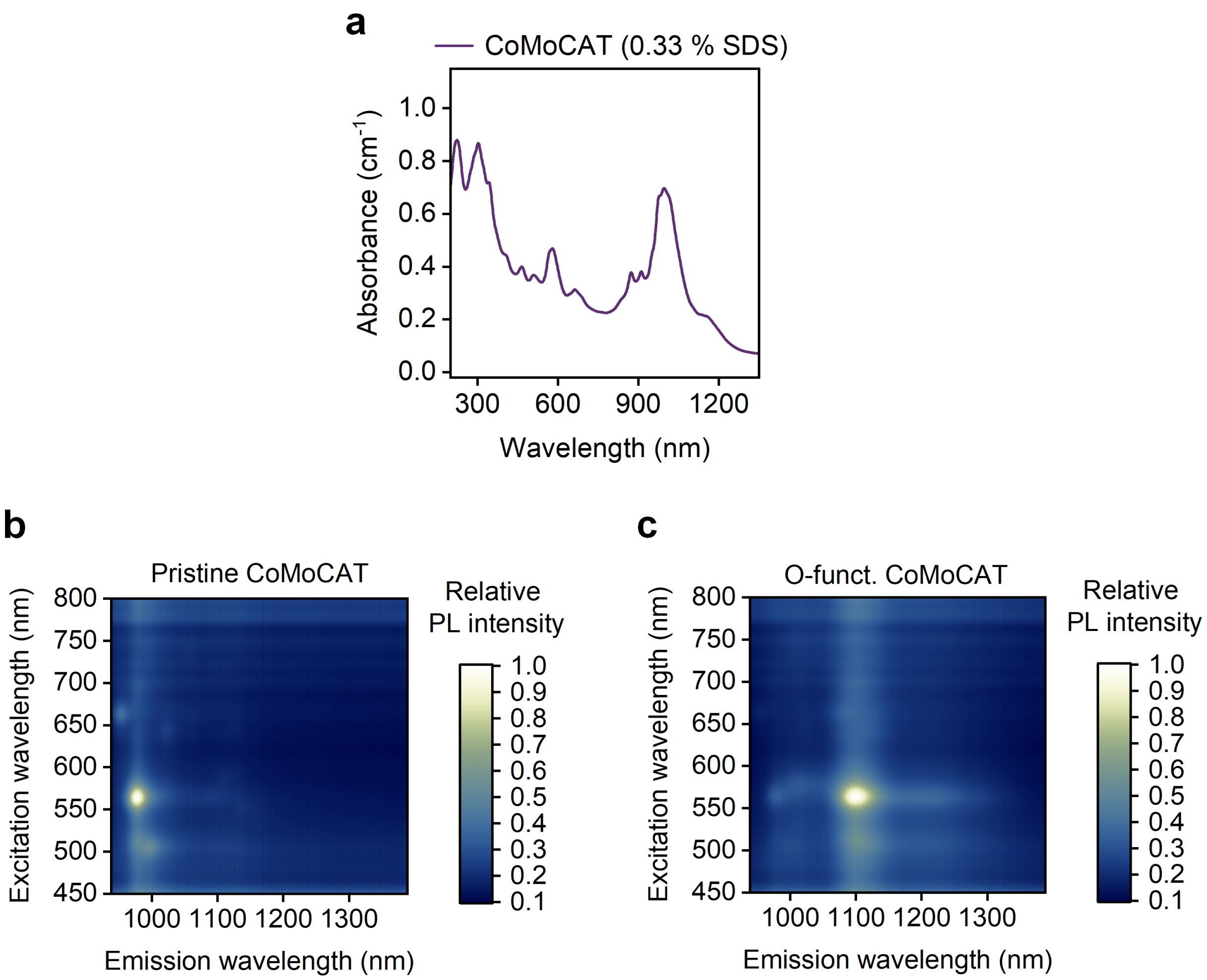

Supplementary Fig. 17 | Functionalization of CoMoCAT raw material. a UV-Vis-NIR absorption spectrum of CoMoCAT raw material dispersed in 0.33% (w/v) SDS. **b** PLE map of CoMoCAT raw material dispersed in 0.33% (w/v) SDS. **c** PLE map of oxygen-functionalized CoMoCAT raw material (0.33% w/v SDS).

Suppl. Fig. 18 | Tip-sonicated SWCNTs

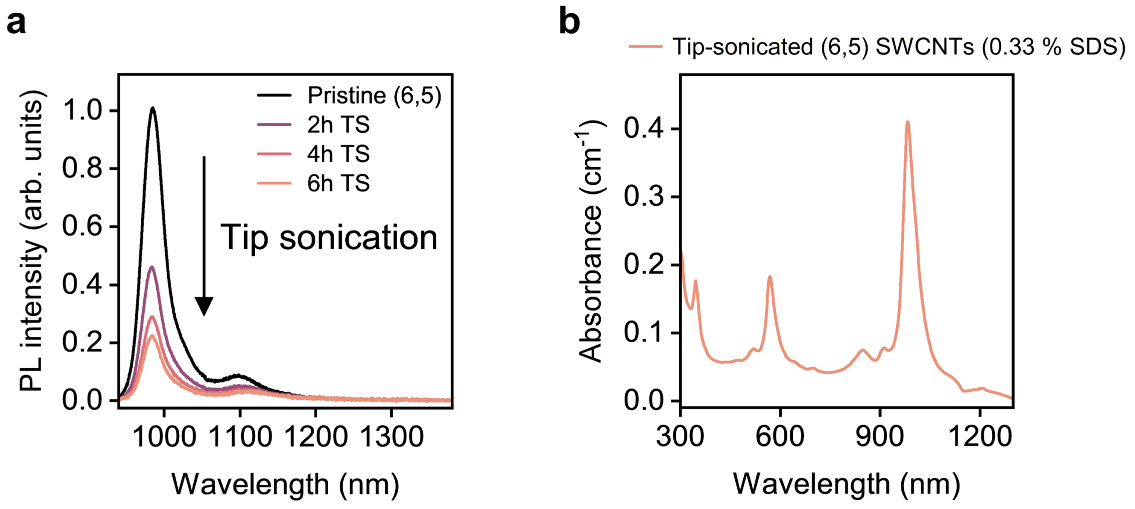

Supplementary Fig. 18 | Shortening of SWCNTs by tip sonication. a PL spectra of (6,5) SWCNTs (1% w/v SDS) after different durations of tip sonication (TS). **b** UV-Vis-NIR absorption spectrum of (6,5) SWCNTs after 6 h of tip sonication (1% w/v SDS).

Suppl. Fig. 19 | AFM length statistics

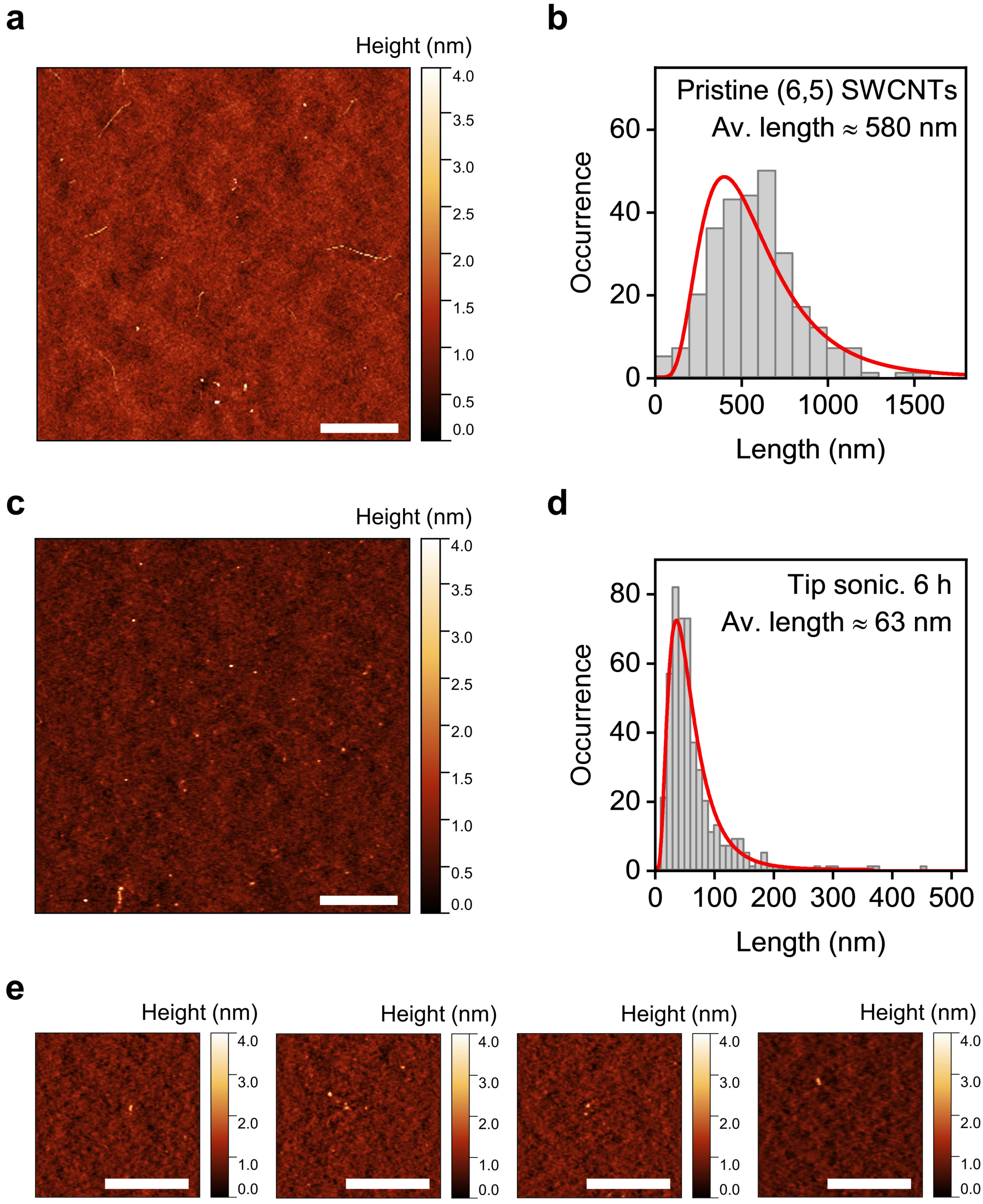

Supplementary Fig. 19 | AFM length statistics of tip-sonicated SWCNTs. a Representative atomic force micrograph of pristine (6,5) SWCNTs (scale bar, 1 µm). **b** Length histogram of pristine (6,5) SWCNTs with an average length of 580 nm (log-normal distribution, red curve. **c** Representative atomic force micrograph of (6,5) SWCNTs after 6 h of tip sonication (scale bar, 1 µm). **d** Length histogram of (6,5) SWCNTs after 6 h of tip sonication with an average length of 63 nm (log-normal distribution, red curve). **e** Zoomed-in atomic force micrographs (1 µm × 1 µm) of shortened (6,5) SWCNTs (scale bar, 500 nm).

Suppl. Fig. 20 | Organic solvent SWCNT dispersions

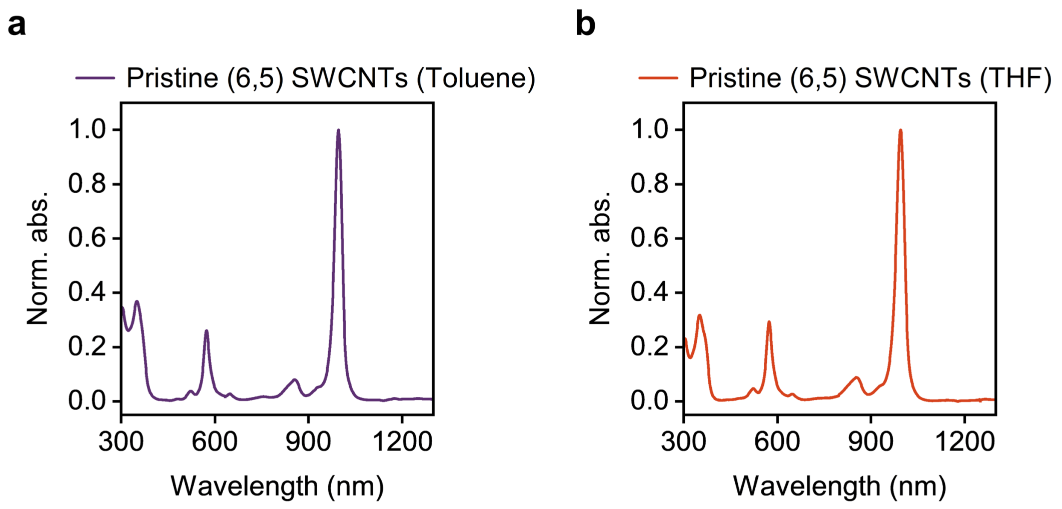

Supplementary Fig. 20 | Absorption spectra of (6,5) SWCNT dispersions in organic solvents. Normalized UV-Vis-NIR absorption spectra of PFO-BPy-wrapped (6,5) SWCNTs in toluene (**a**) or THF (**b**).

Suppl. Fig. 21 | PLE maps of organic solvent SWCNT dispersions

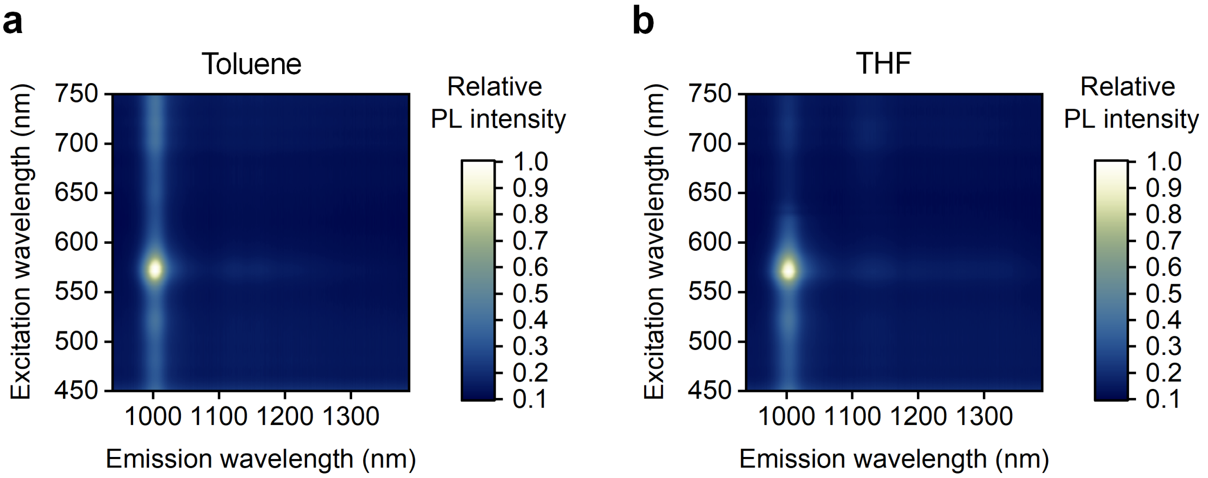

Supplementary Fig. 21 | SWCNT dispersions in organic solvents. PLE maps of PFO-BPy-wrapped (6,5) SWCNTs in toluene (**a**) and THF (**b**).

Suppl. Fig. 22 | Treatment of (6,5) SWCNTs with *t*-Bu-AQ in toluene

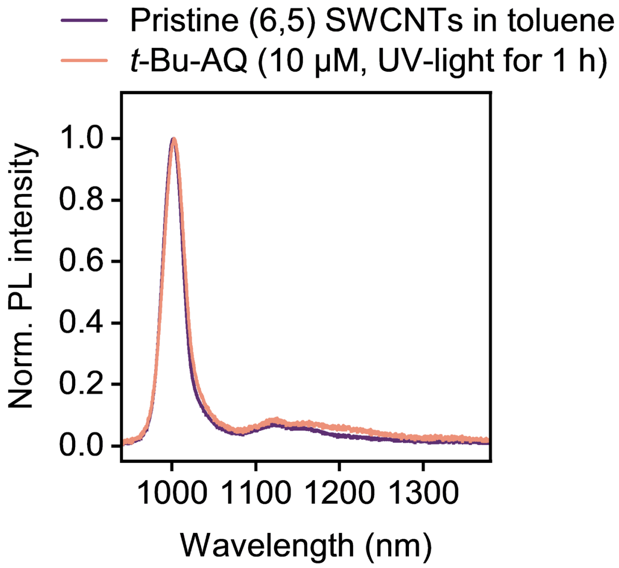

Supplementary Fig. 22 | Treatment of toluene dispersions of (6,5) SWCNTs with *t*-Bu-AQ. PL spectra of (6,5) SWCNTs (wrapped by PFO-BPy in toluene) before and after treatment with *t*-Bu-AQ and UV-light (365 nm) for 1 h.

Suppl. Fig. 23 | Single-nanotube PL spectra at cryogenic temperature

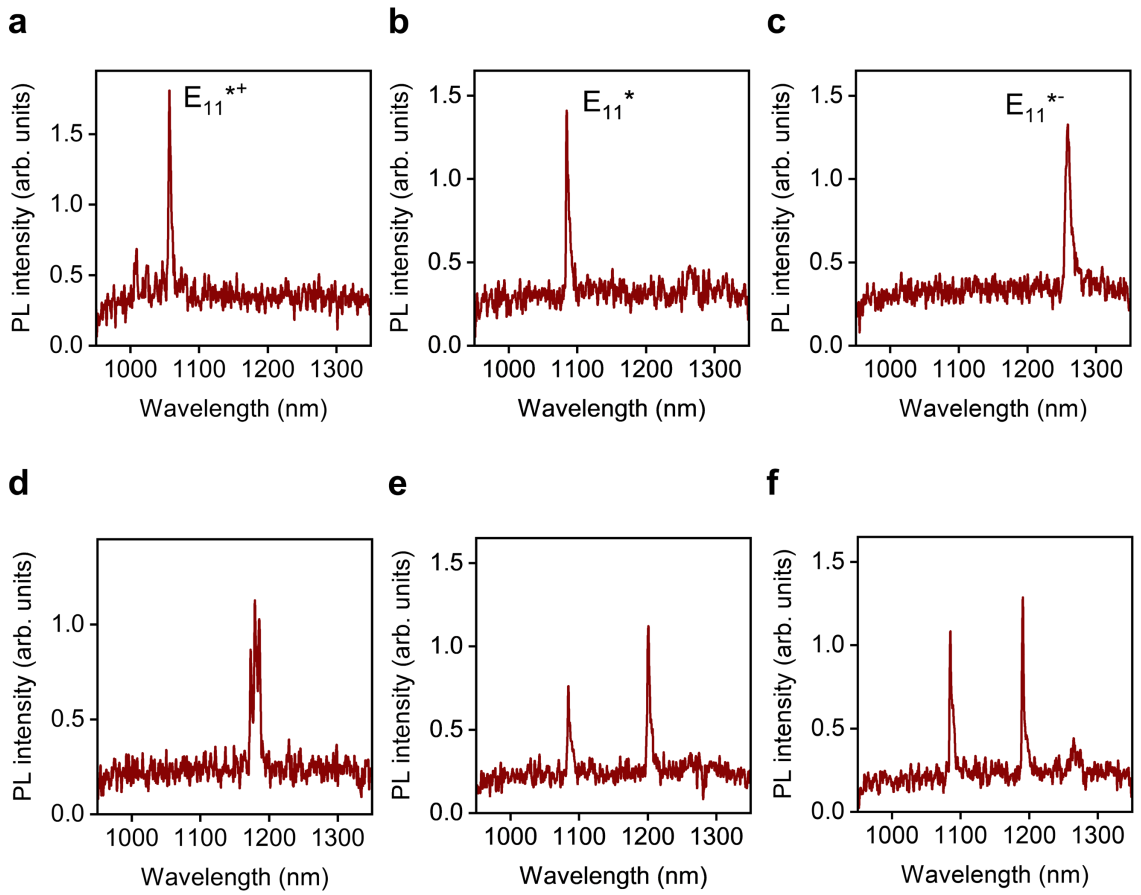

Supplementary Fig. 23 | Low-temperature (4.6 K) single-SWCNT spectra of PFO-BPy-wrapped (6,5) SWCNTs functionalized using 2-*tert*-butylanthraquinone in THF. Single-nanotube PL spectra with E_{11}^{*+} (**a**), E_{11}^{*} (**b**) and E_{11}^{*-} (**c**) emission peaks[5]. **d, e, f** Single-nanotube PL spectra with spectral signatures of multiple defects.

Suppl. Fig. 24 | Spatial resolution of film functionalization

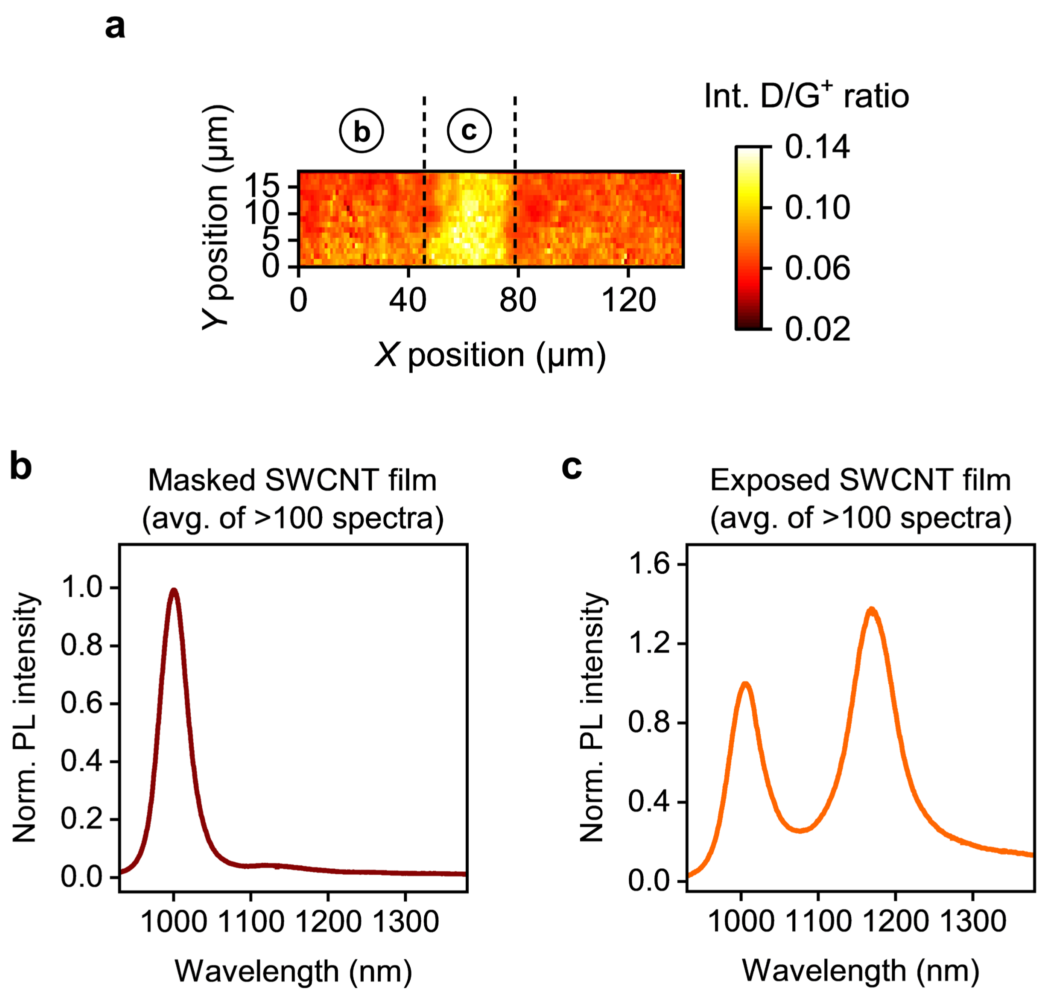

Supplementary Fig. 24 | Spatial resolution of photocatalytic SWCNT film functionalization. a Map of integrated Raman D/G$^+$ ratio of a thin-film of PFO-BPy-wrapped (6,5) SWCNTs after photocatalytic functionalization with aqueous AQS solution (2 mg mL^{-1}) and UV irradiation (365 nm, 1 h). The employed shadow mask consisted of a line structure (width 50 µm). Average PL spectra of masked (**b**) and exposed area (**c**) after functionalization (>100 randomly sampled positions for each area).

Suppl. Fig. 25 | Absorption spectra of SWCNT enantiomers

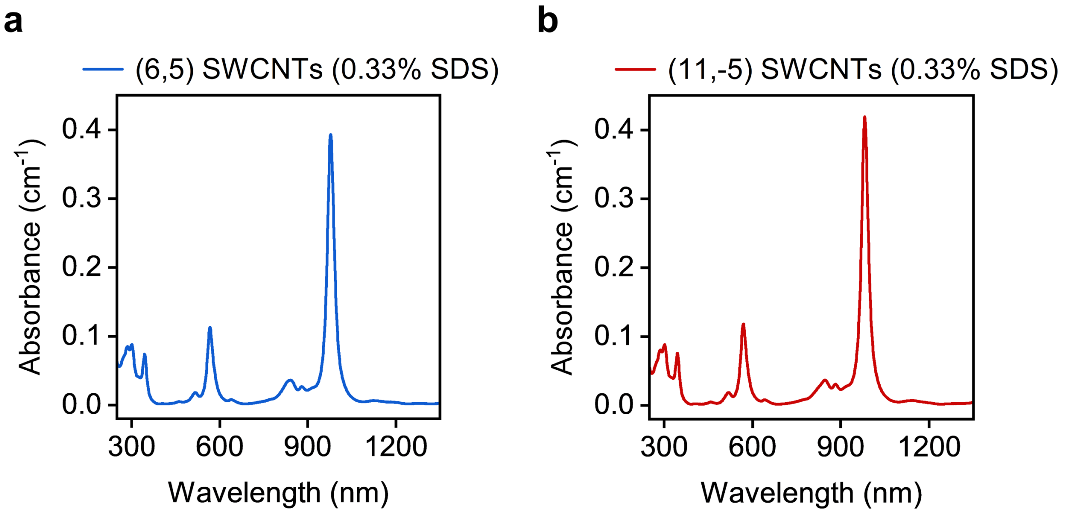

Supplementary Fig. 25 | Absorption spectra of enantiopure SWCNTs. UV-Vis-NIR absorption spectra of aqueous dispersions of enantiopure (6,5) (**a**) and (11,-5) (**b**) SWCNTs.

Suppl. Fig. 26 | PLE maps of SWCNT enantiomers

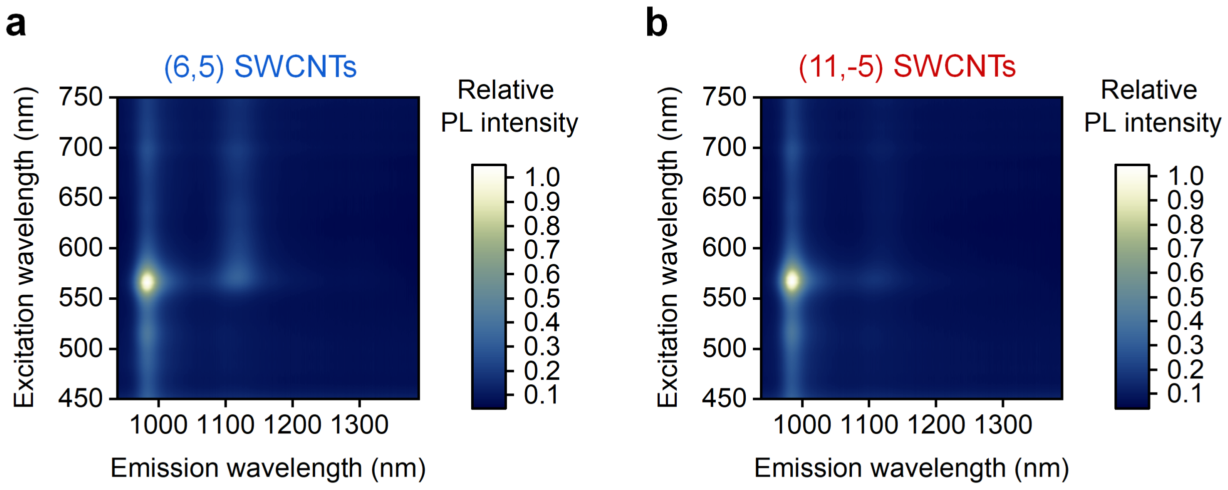

Supplementary Fig. 26 | PLE maps of SWCNT enantiomers. Photoluminescence excitation-emission maps of (6,5) (**a**) and (11,-5) (**b**) SWCNTs (aqueous dispersions). Note, the (6,5) SWCNT dispersion in (**a**) shows weak emission from a few unintentional luminescent defects. Due to their low density, they do not affect the absorption or CD spectra.

Suppl. Fig. 27 | UV-Vis CD spectra of functionalized SWCNTs

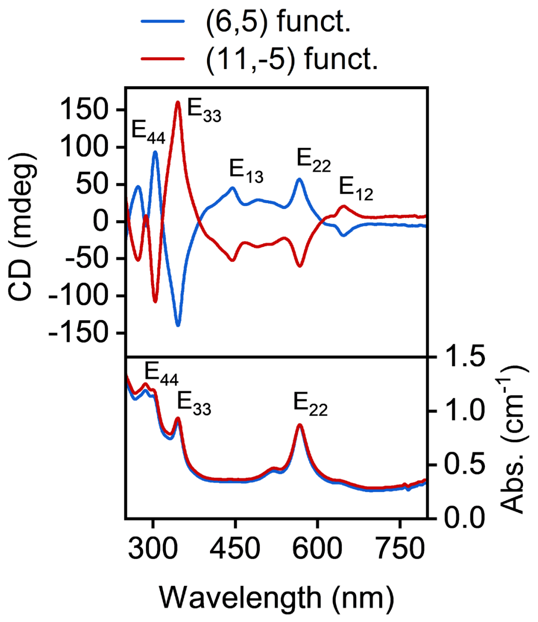

Supplementary Fig. 27 | CD and absorption spectra of functionalized SWCNT enantiomers. Circular dichroism and UV-Vis absorption spectra (250 – 800 nm) of oxygen-functionalized (6,5) and (11,-5) SWCNT enantiomers (in aqueous dispersion, 1% w/v DOC).

Suppl. Fig. 28 | Raman spectra of SWCNT enantiomers

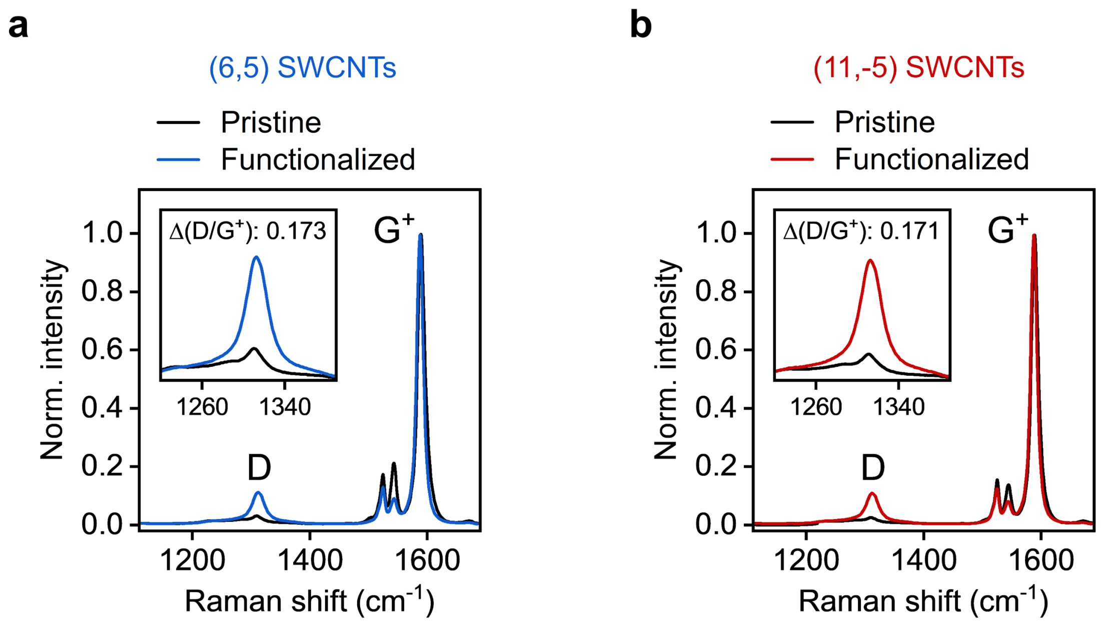

Supplementary Fig. 28 | Raman spectroscopy of pristine and functionalized SWCNT enantiomers. Normalized and averaged (>3000 spectra) Raman spectra (λ_{exc} = 532 nm) of pristine and highly functionalized (6,5) (**a**) and (11,-5) (**b**) SWCNTs as used for circular dichroism measurements. Insets show the D-mode and integrated $\Delta(D/G^{+})$ ratio.

Supplementary Note 1| Influence of exciton trapping on absorption dissymmetry factor

The absorption dissymmetry factor g_{abs} (or equivalently, the normalized circular dichroism signal $\mathrm{CD_{norm}}$) of an optical transition can be calculated from the rotational strength R and dipole strength D of a molecule according to

$$g_{\mathrm{abs}} = \frac{4R}{D}, \tag{4}$$

where

$$R = |\mu||m| \cos\theta \tag{5}$$

and

$$D = |\mu|^2 + |m|^2. \tag{6}$$

μ and m denote the electronic and magnetic transition dipole moments, respectively, which include the angle θ.[6] For alternating E_{ii} transitions in SWCNTs (i = 1, 2, 3…), the orientation of μ and m changes, resulting in opposite signs for odd and even E_{ii} transitions in CD spectra of single-enantiomer SWCNTs[7] (see also Supplementary Fig. 27). In contrast to that, the CD signals of the E_{11} and E_{11}* optical transitions have the same sign, indicating that the orientation of the transition dipole moments is not reversed for free and defect-localized excitons (see Fig. 4c of the main text).

For a comparative, semi-quantitative estimate of absorption dissymmetry factors g_{abs} of the E_{11} and E_{11}* transitions, we assume that exciton localization at a luminescent defect site leads to a reduction in exciton size, while the symmetry of the excitonic wavefunction remains unchanged. In agreement with previous TD-DFT simulations[5,8], the magnitude of the interaction with the nanotube lattice is assumed to remain identical for free and defect-localized exciton, as the respective electron and hole densities are strongly confined on the nanotube lattice (instead of the attached functional group or atom defect). A reduction in exciton size due to trapping at a luminescent defect results in a three- to four-fold reduction in oscillator strength f_{12},[8] which is related to the electronic transition dipole moment μ_{12} for a transition between two states $|1\rangle$ and $|2\rangle$ according to

$$f_{12} = \frac{2}{3}\Delta E_{12}\,|\mu_{12}|^2. \tag{7}$$

Here, ΔE_{12} denotes the difference in energy between the initial and final states $|1\rangle$ and $|2\rangle$. Consequently, for the E_{11} and E_{11}* optical transitions, a difference in electronic transition dipole

moments of ≈ 1.7 – 1.9 is found from the aforementioned difference in oscillator strength. Based on theoretical work on SWCNTs and data from synthesized nanotube segments[9-11], μ and m can be estimated to be on the order of 1 D and 0.1 μ_B, which are typical values found in helically chiral organic molecules with comparable absorption dissymmetry factors as observed for the E_{11} transition in SWCNTs (~ 10^{-4} – 10^{-3})[12-14]. Any change in magnetic transition dipole moment between the E_{11} and E_{11}* state is assumed to be negligible, as they are practically identical in geometry[11,12].

Overall, these values result in an estimate for g_abs of ≈ $4 \cdot 10^{-4}$ and ≈ $2 \cdot 10^{-4}$ for the E_{11} and E_{11}* absorption, respectively, which aligns well with our findings ($g_\mathrm{abs}(E_{11}) \approx 9 \cdot 10^{-4}$ and $g_\mathrm{abs}(E_{11}^*) \approx 5 \cdot 10^{-4}$). Thus, we conclude that a reduction in exciton size by a factor of ≈ 2 – 3, accompanied by a proportional decrease in oscillator strength and electronic transition dipole moment, results in the observed reduction of the absorption dissymmetry factor from the E_{11} to the E_{11}* optical transition, which in turn enables the direct calculation of relative exciton sizes based on CD spectroscopy.

Supplementary References

1. Sebastian, F. L. et al. Absolute quantification of sp^3 defects in semiconducting single-wall carbon nanotubes by Raman spectroscopy. *J. Phys. Chem. Lett.* **13**, 3542-3548 (2022).

2. Sebastian, F. L. et al. Unified quantification of quantum defects in small-diameter single-walled carbon nanotubes by Raman spectroscopy. *ACS Nano* **17**, 21771-21781 (2023).

3. Hartmann, N. F. et al. Photoluminescence dynamics of aryl sp^3 defect states in single-walled carbon nanotubes. *ACS Nano* **10**, 8355-8365 (2016).

4. Kim, M. et al. Fluorescent carbon nanotube defects manifest substantial vibrational reorganization. *J. Phys. Chem. C* **120**, 11268-11276 (2016).

5. Ma, X. et al. Electronic structure and chemical nature of oxygen dopant states in carbon nanotubes. *ACS Nano* **8**, 10782-10789 (2014).

6. Covington, C. L. & Polavarapu, P. L. Similarity in dissymmetry factor spectra: a quantitative measure of comparison between experimental and predicted vibrational circular dichroism. *J. Phys. Chem. A* **117**, 3377-3386 (2013).

7. Wei, X. et al. Experimental determination of excitonic band structures of single-walled carbon nanotubes using circular dichroism spectra. *Nat. Commun.* **7**, 12899 (2016).

8. Gifford, B. J., Kilina, S., Htoon, H., Doorn, S. K. & Tretiak, S. Exciton localization and optical emission in aryl-functionalized carbon nanotubes. *J. Phys. Chem. C* **122**, 1828-1838 (2018).

9. Motavas, S., Ivanov, A. & Nojeh, A. Optical transitions in semiconducting zigzag carbon nanotubes with small diameters: a first-principles broad-range study. *Phys. Rev. B* **82**, 085442 (2010).

10. Sato, S. et al. Chiral intertwined spirals and magnetic transition dipole moments dictated by cylinder helicity. *Proc. Natl. Acad. Sci. U.S.A.* **114**, 13097-13101 (2017).

11. Kogashi, K., Matsuno, T., Sato, S. & Isobe, H. Narrowing segments of helical carbon nanotubes with curved aromatic panels. *Angew. Chem. Int. Ed.* **58**, 7385-7389 (2019).

12. Qiu, Z. et al. Amplification of dissymmetry factors in π-extended [7]- and [9]helicenes. *J. Am. Chem. Soc.* **143**, 4661-4667 (2021).

13. Xu, F. et al. Supramolecular polymerization as a tool to reveal the magnetic transition dipole moment of heptazines. *J. Am. Chem. Soc.* **146**, 15843-15849 (2024).

14. Tanaka, H., Inoue, Y. & Mori, T. Circularly polarized luminescence and circular dichroisms in small organic molecules: correlation between excitation and emission dissymmetry factors. *ChemPhotoChem* **2**, 386-402 (2018).
