# Supplementary material for: Circular dichroism of quantum defects in carbon nanotubes created by photocatalytic oxygen functionalization
Source: Nat Commun. 2025 Jun 2;16:5107. doi: 10.1038/s41467-025-60342-y (PMC12130195; doi:10.1038/s41467-025-60342-y)
Supplement: Supplementary file 2 — Description of Additional Supplementary Files [file 41467_2025_60342_MOESM2_ESM.pdf]

Description of Additional Supplementary Files

File Name: Supplementary Movie 1

Description: Simultaneous functionalization and PL imaging of (6,5) SWCNT dispersions. Two vials with aqueous (6,5) SWCNT dispersions (identical concentrations) with 4 µM AQS (left) and without AQS (right) under continuous irradiation by UV light (365 nm) and simultaneous imaging of near-infrared emission (950 – 1600 nm) over 120 min.
